# Neighborhood, Peer, and Parental Influences on Minor and Major Substance Use of Latino and Black Adolescents

**DOI:** 10.3390/children8040267

**Published:** 2021-03-31

**Authors:** Marika Sigal, Bryan J. Ross, Andrew O. Behnke, Scott W. Plunkett

**Affiliations:** 1 Department of Psychology, California State University Northridge, 18111 Nordhoff St., Northridge, CA 91330, USA; marika.sigal.242@my.csun.edu (M.S.); bryan.ross.529@my.csun.edu (B.J.R.); 2 School of Family and Consumer Sciences, Texas State University, 601 University Drive, San Marcos, TX 78666, USA; aob28@txstate.edu

**Keywords:** adolescent, Black, Latino, substance use, neighborhood, friends’ delinquency, peer victimization, parental monitoring

## Abstract

Self-report survey data were collected from 797 adolescents (47.2% Latino, 52.8% Black) in North Carolina. Path analyses were conducted to examine relationships between youth perceptions of maternal and paternal monitoring, neighborhood crime/drugs, friends’ delinquency, peer victimization, minor substance use, and major substance use. After establishing a good fitting model, multigroup models were conducted for Blacks vs. Latinos. The results indicated perceived maternal monitoring (and paternal monitoring for Latinos) was directly related to decreased exposure to neighborhood crime/drugs and friends’ delinquency. For Latinos and Blacks, maternal and paternal monitoring were directly related to gateway substance use, and indirectly related to major substance use through gateway substance use. Additionally, friends’ delinquency and peer victimization were directly related to gateway and major substance use for Blacks and Latinos. Thus, exposure to neighborhood crime/drugs was indirectly related to substance use through friends’ delinquency and peer victimization.

## 1. Introduction

**Figure 1:**
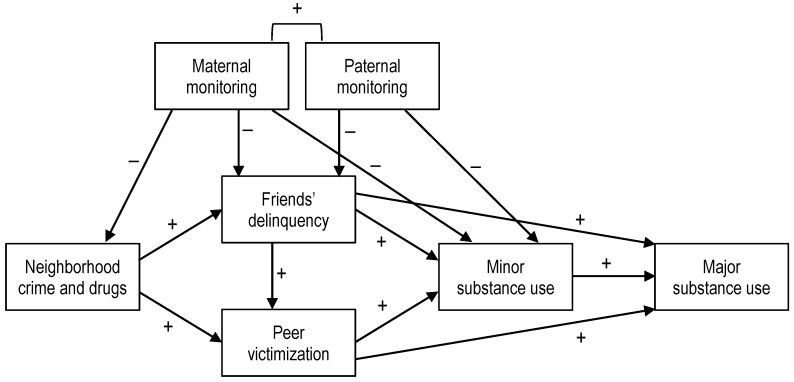
Theoretical model examining neighborhood, peer, and parenting antecedents of substance use in Latino and Black adolescents.

A major mental and physical health concern for adolescents is substance use. Although substance use is often examined in isolation (e.g., just alcohol or just cannabis use), studies have repeatedly shown evidence for polysubstance use (e.g., youth who engage in alcohol use will often engage in tobacco and/or cannabis [1]). Furthermore, studies have shown that minor substances (e.g., alcohol, tobacco, cannabis) increase risk of major substances (e.g., cocaine [1,2]). Both minor and major drug use increase risk or co-occur with mental health issues (e.g., depression [1,2,3]). Thus, it is important to identify factors that make some adolescents more prone to engaging in risky behaviors such as substance use [4]. Specifically, exposure to substance use in the community and within peer groups increases the likelihood that adolescents will use substances [5,6]. However, monitoring by parents can decrease perceptions of neighborhood risks [7], adverse peer influences [8], and substance use [9]. The purpose of this study was to investigate neighborhood risks, peer influences (i.e., delinquency, victimization), and maternal and paternal monitoring on minor and major substance use of Latino and Black adolescents in North Carolina (see Figure 1).

### 1.1. Adolescent Substance Use

Nationwide, 60.4% of adolescents reported ever consuming alcohol, and 35.6% reported ever using cannabis [4]. A 2017 survey of high school students found that 32.4% of White, 31.3% of Latino, and 20.8% of Black high school students currently drank alcohol, and 31.8% of the females and 27.6% of the males currently drank alcohol [4]. Latino and Black adolescents were more likely to have had their first drink before the age of 13 than their White peers (i.e., 19.3% Latinos; 14.9% Blacks; 14.0% Whites [4]). In addition, 42.8% of Black high school students and 42.4% of Latino high school students reported cannabis use compared to 32.0% of White students [4].

Studies have found the use of minor substances (e.g., tobacco, alcohol, cannabis) increases the likelihood of using major substances (e.g., cocaine, methamphetamine; [1,2,10]). This relationship is sometimes explained by gateway theory which identifies a prevalent link between use of minor substances in childhood and adolescence as predictors of later hard-drug use [10,11,12]. For example, developmental trajectories of early alcohol use show later alcohol dependence as well as significantly increased risk for substance use [13]. This is especially concerning since alcohol is a legal and readily available substance in the United States. Research has also identified tobacco as a gateway substance to cannabis or other drugs [10,14]. In a twin study, a twin who reported using cannabis before the age of 17 had up to 3.9 increase in probability of other drug use and up to 6.0 increase in probability of addiction to and abuse of alcohol and other drugs versus the other twin who did not try cannabis [15]. Another study reported cannabis users were two times more likely to use illicit drugs than non-users [16]. These findings suggested there was a substantial increase in the willingness to try major substances if prior experience with a minor substance was present. Moreover, the strength of the relationship between minor substances and major substance use remains significant even after controlling for multiple factors (e.g., environmental risk, history of behavioral problems, age, gender, race [16]).

### 1.2. Contextual Risk Factors of Adolescents’ Substance Use

Various theories have been used to explain contextual risk factors for adolescent substance use [17]. Socialization theory suggests that youth who associate with and/or see others (e.g., neighbors, peers) engaging in substance use are more likely to see substance use as normalized, and thus, have increased likelihood of trying and using substances [17,18,19]. Social learning theory suggests substance use is a behavior learned through modeling, imitation, vicarious reinforcement, and differential association-reinforcement [17,20]. For example, substance use might be vicariously reinforced when community members or peers are perceived as experiencing benefits from using substances (e.g., feeling good, fitting in with others). Personality traits of the youth might influence the socialization process, thus impacting the risk of substance use [19]. Self-derogation theory suggests youth might use substances to cope when their self-esteem suffers due to hostile and/or stressful environments (e.g., disadvantaged neighborhoods, peer victimization [17,21]).

Studies have found that neighborhoods significantly encourage engagement in social activities, especially for adolescents [22]. Thus, regularly witnessing substance use and having it readily available can increase the likelihood of use, can encourage normalization of these behaviors, and can contribute to later substance use in Latino and Black adolescents [22,23,24]. Research found that high visibility of drug sales was the biggest disadvantage of densely populated neighborhoods for Black youths [23]. When adolescents perceived more availability in their neighborhoods, they were more likely to view substance use as normal (i.e., socialization theory) and/or model the behaviors (i.e., social learning theory). Furthermore, perceived availability of alcohol in the neighborhood was positively related to current and future alcohol use by early adolescents [25]. In addition, higher neighborhood drug prevalence was related to increased risk of cannabis use among a mostly Black sample of adolescents and emerging adults [24].

Various theories have suggested why friends’ delinquent behaviors are related to adolescents’ substance use. For example, socialization theory proposes that adolescents may engage in behaviors observed by significant others, such as peers [17]. When peers have substances and/or give substances to their friends, then they will be more likely to use substances. For example, a nationwide survey found that 43.5% of high school students (i.e., 48.4% female and 37.8% male) usually drank alcohol given to them by someone else [4]. Further, social learning theory proposes that adolescents’ behaviors are shaped through positive reinforcement [17]. For example, some adolescents may use substances to increase their popularity [5]. 

Peer victimization is defined as aggressive nonsexual behavior experienced by youths from their peers [26]. Studies have found an increased risk of substance use after experiencing peer victimization [27,28]. As previously stated, self-derogation theory suggests that youth with low self-esteem from hostile or stressful environments may use substances to cope [17]. There seems to be evidence to support this idea since peer victimization is related to low self-esteem and depression [26]. Furthermore, bullying can lead to using alcohol as a coping strategy [6]. Thus, peer victimization seems to be implicated in adolescent substance use.

### 1.3. Contextual Risk Factors of Adolescents’ Substance Use

Various parenting styles and also specific parental behaviors have been examined in relation to adolescents’ substance use [9,29]. Of all the parenting dimensions, parental monitoring has been consistently identified as a key parental contributor to adolescents’ substance use [9,30] as well as contextual risk factors [7,8]. Parental monitoring (e.g., supervision and/or knowledge of children’s activities) is an important component of parents’ behavioral control of the adolescent [31]. However, parental monitoring is conveyed and/or perceived differently from culture to culture. In Black and Latino families, monitoring may be perceived as a form of support [31]. For example, in Latino families, monitoring may be viewed as “protectiveness” and supporting the cultural notions of maintaining strong family ties and respect for one another [31]. 

When parents know what their children are doing, they may be less likely to use substances [30]. Parental monitoring has been found to relate to lower adolescent substance use [32,33]. Specifically, parental monitoring was negatively related to adolescents’ binge drinking behavior [29], alcohol misuse [32], cannabis use (see meta-analyses [30]), and drug use [34]. Studies with minority samples found that perceived parental monitoring was related to decreased cannabis use by Latino adolescents [35], lower rates of drinking alcohol and smoking cannabis by Latino adolescents [36], decreased use of cigarettes, cannabis, and inhalants by Mexican-origin adolescents [37], and lower use of alcohol and cigarettes by Latino preadolescent boys and girls and lower cannabis use by girls [38]. Given that parental monitoring can decrease exposure to neighborhood risks, associating with delinquent peers, and peer victimization, monitoring by mothers and fathers may be indirectly related to substance use. Thus, direct and indirect paths will be examined in the model (see Figure 1).

## 2. Materials and Methods

### 2.1. Subsection

This study was approved by a university institutional review board prior to data collection. Data from Black and Latino students were collected in three North Carolina schools: a rural school, an inner-city school, and a suburban school. First, written permission from an administrator at each school was secured. Then teachers (recommended by the administrators) were asked to help. Researchers went to 9th and 10th grade classrooms to recruit participants. The researchers explained the research topic, and then packets were given to students that included a survey, an adolescent assent form, and a parental consent form. The surveys were in English, while the parental consent forms were in English and Spanish. Students were instructed to first get signed parental consent, then sign the youth assent form, then complete the survey, and finally return the forms and survey to their teacher. Students were instructed to put the surveys in a sealed envelope prior to returning the survey to the teacher. If a student returned a survey along with signed assent and consent forms, then a $15 gift card was given to the student as an incentive. Teachers were also compensated with a gift card for their help. 

Surveys were coded by trained research assistants (RAs), then the coding was double-checked by different RAs. Next, the coded surveys were entered into an Excel file by the RAs, and then the data entry was double-checked by other trained RAs. Next, frequencies were run on all variables as one more check for accuracy.

The total sample consisted of 376 Black adolescents and 421 Latino adolescents (45.8% boys and 54.2% girls). Sample characteristics are shown in Table 1. The Black adolescents were slightly but significantly older than the Latino adolescents (*t* = 2.43, *p* = 0.015, *M_diff_* = 0.22). Similarly, there was a significant ethnic group difference between grade classifications (χ^2^ = 45.16, *p* < 0.001), demonstrated by more 12th graders who were Black than Latino. This finding is due to the classroom teachers who agreed to participate (i.e., there were more 12th grade teachers who participated at a school that had more Black than Latino students). No significant ethnic group differences were found on gender (χ^2^ = 0.47, *p* = 0.494). Furthermore, there were significant ethnic group differences in family structure (χ^2^ = 48.96, *p* < 0.001) with a much higher percentage of Latino youth in two-parent intact families, and a much higher percentage of Black youth in single mother families, stepfather families, and other family forms. These findings are consistent with much literature on Latino and Black family structures. As expected, there were significant ethnic group differences on generational status (χ^2^ = 667.99, *p* < 0.001) with a much higher percentage of Latino youth from immigrant families.

### 2.2. Measures

Sample characteristics were measured with standard demographic items. To assess ethnic group, participants were asked an open-ended question (i.e., “What is your ethnicity or race?”) Participants were included in the Black subsample if they answered African American, Afro-American, or Black. Participants were included in the Latino sample if they answered Hispanic, Chicano/a, Latino/a/x, or a Latin American country/region (e.g., Salvadoran, Guatemalan, Cuban, Central American). The overall survey took approximately 30–45 min to complete. The scales used in the study are outlined below. For the scales, the items were averaged to create scale scores.

An 8-item scale was used to measure minor substance use (4 items) and major substance use (4 items) [7]. The stem for the items follows: “In the past six months how often have you…” The minor substance use items were: (a) “Smoked cigarettes,” (b) “Drank alcohol (beer, wine, hard liquor),” (c) “Gotten drunk,” and (d) “Used marijuana/pot.” The major substance use items were: (a) “Used speed, meth, or cocaine (crack, cheese)” (b) “Used inhalants or sniffed (noz, glue, cleaners),” (c) “Used acid, LSD, ecstasy, PCP, mushrooms,” and (d) “Used prescription drugs NOT prescribed by a doctor.” The response choices follow: 0 = never, 1 = once, 2 = a few times, and 3 = many times. In the current data, the Cronbach’s alphas for minor substance use were 0.82 for Blacks and 0.87 for Latinos, and for major substance use the alphas were 0.91 for Blacks and 0.87 for Latinos.

A 5-item version of the neighborhood risks scale was used to measure neighborhood risks related to illegal activities and substance use [39]. Participants were asked the following five items: (a) “I have seen people do illegal things,” (b) “There is a lot of crime,” (c) “There is a lot of violence,” (d) “Many people use drugs or drink alcohol,” and (e) “Illegal drugs are readily available.” The response options were: 1 = strongly disagree, 2 = disagree, 3 = agree, and 4 = strongly agree. In the current data, the Cronbach’s alphas were 0.93 for Blacks and 0.92 for Latinos.

A 7-item scale was used to measure adolescents’ friends’ delinquent behaviors (e.g., gang involvement, vandalism, substance use) within the past 30 days. The scale was modified from one developed by the Center for Urban Affairs and Policy Research [40]. The response options were: 0 = never, 1 = sometimes, 2 = frequently, 3 = very frequently, and 4 = always. In the current data, the Cronbach’s alphas were 0.89 for Blacks and 0.90 for Latinos.

A 9-item scale was used to measure peer victimization [41]. The following stem was used: “In the last 6 months how often has another kid…” Sample items follow: “Called me mean names to hurt my feelings” and “Threatened me.” The response choices were: 1 = never, 2 = once or twice, 3 = sometimes, 4 = once a week, and 5 = more than once per week. In the current data, the Cronbach’s alphas were 0.89 for Blacks and 0.94 for Latinos.

The 6-item monitoring subscale was used from the Parent Behavior Measure [42]. A sample items follow: “I tell this parent who I am going to be with when I go out.” The response options were: 1 = strongly disagree, 2 = disagree, 3 = agree, and 4 = strongly agree. In the current data, the Cronbach’s alphas for mothers’ monitoring were 0.82 for Blacks and 0.84 for Latinos, and the alphas for fathers’ monitoring were 0.94 for Blacks and 0.91 for Latinos.

### 2.3. Data Analyses

First, bivariate correlations were conducted among the primary variables to be used in the regressions. Next, path analyses were conducted using the Lavaan package in R [43]. First, one path model was fit across the entire sample. Next, we fit separate models grouping by genders and ethnic groups, respectively. For both genders and ethnicity, two models were initially fit to determine any potential differences in paths: one in which all paths were allowed to vary, and one in which all paths were constrained. Chi-squared difference tests were conducted to test for significant differences between models. If significant differences were found, individual paths were then sequentially constrained, with each new model again being compared against the baseline—i.e., the model with no constraints. Lastly, a final model was fit with all constraints not associated with significant model differences and again tested against the baseline model. A non-significant difference indicated appropriate constraints and varying paths were in place. All models were fit using maximum likelihood estimation, and model performance was evaluated based upon a comparative fit index (CFI) ≥ 0.95, and a root mean square error of approximation (RMSEA) ≤ 0.05 [44].

## 3. Results

Means, standard deviations, and correlations for male and female adolescents are shown in Table 2, while means, standard deviations, and correlations for Blacks and Latinos are shown in Table 3. For all subsamples, minor substance use was significantly and positively correlated to major substance use. Furthermore, neighborhood risks, peer victimization, and friends’ delinquency were significantly and positively correlated to major and minor substance use in each subsample. Neighborhood risks were significantly and positively correlated to peer victimization and friends’ delinquency in each subsample. Peer victimization and friends’ delinquency were significantly and positively correlated in each subsample.

For all subsamples, maternal monitoring was related to minor substance use, neighborhood risks, and friends’ delinquency. For Latinos, maternal monitoring was also significantly related to major substance use. For boys and Latinos, paternal monitoring was significantly and negatively correlated to all the variables. For girls, paternal monitoring was only significantly related to minor substance use and friends’ delinquency. For Blacks, paternal monitoring was significantly related to minor substance use, neighborhood risks, and friends’ delinquency.

**Figure 2:**
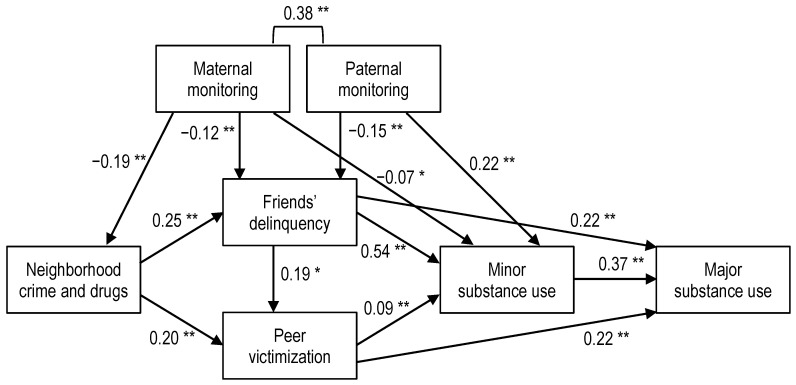
Empirical model showing neighborhood, peer, and parenting antecedents of substance use in Latino and Black adolescents. Standardized betas are shown. Chi-square = 24.60, *p* < 0.001, comparative fit index (CFI) = 0.982, root mean square error of approximation (RMSEA) = 0.06. * *p* < 0.05. ** *p* < 0.01.

Fit indices for the total sample indicated the model was an excellent fit to the data: χ^2^(7, 689) = 24.60, *p* < 0.001; SRMR = 0.03; RMSEA = 0.06 (90% CI (0.04, 0.09)); CFI = 0.98. The standardized path coefficients are shown in Figure 2.

For the first multiple-group model on genders, all paths were free to vary. This model fit the data well: χ^2^(14, 689) = 32.26, *p* = 0.004; SRMR = 0.03; RMSEA = 0.06 (90% CI (0.03, 0.09)); CFI = 0.98. The model in which all paths were constrained did poorly in comparison: χ^2^(34, 689) = 92.89, *p* < 0.001; SRMR = 0.06; RMSEA = 0.07 (90% CI (0.05, 0.09)); CFI = 0.94 and performed significantly worse χ^2^_diff_ (20) = 60.63, *p* < 0.001. This indicated differences in paths between genders and that coefficients needed to be interpreted individually. Each path was then individually constrained and tested against the model with freely varying parameters. Significant differences between the two indicated worse model fit from the constraint, and thus, suggested gender differences on that path. As shown in Table 4, the following gender differences were found: (1) peer victimization and maternal monitoring were significantly related to girls’ minor substance use but not boys’ minor substance use; (2) neighborhood risks were significantly related to girls’ (but not boys’) peer victimization; and (3) paternal monitoring of boys (but not girls) was significantly related to friends’ delinquency. The final model with appropriately constrained and varied paths fit the data well: χ^2^(24, 689) = 47.13, *p* = 0.003; SRMR = 0.05; RMSEA = 0.06 (90% CI (0.03, 0.08)); CFI = 0.98, performing slightly worse than the free-varying model, but not significantly with χ^2^_diff_ (10) = 14.87, *p* = 0.0137.

The same multiple-group methodology used on gender was then used on ethnic groups. The free-varying model fit the data well: χ^2^(14, 689) = 35.03, *p* = 0.001; SRMR = 0.03; RMSEA = 0.07 (90% CI (0.04, 0.09)); CFI = 0.98 in comparison to the fully constrained model χ^2^(34, 689) = 116.58, *p* < 0.001; SRMR = 0.07; RMSEA = 0.08 (90% CI (0.07, 0.10)); CFI = 0.92. This difference was significant χ^2^_diff_ (20) = 81.56, *p* < 0.001; indicating differences in paths between ethnic groups and that coefficients needed to be interpreted individually. As shown in Table 5, ethnic differences were found: (1) friends’ delinquency was significantly related to major substance use for Latinos and Blacks, but it was significantly stronger for Blacks; (2) peer victimization was significantly related to major substance use by Latinos and Blacks, but it was significantly stronger for Latinos; (3) maternal monitoring of Black youth (but not Latino youth) was significantly and negatively related to friends’ delinquency; and (4) paternal monitoring of Latino youth (but not Black youth) was significantly and negatively related to friends’ delinquency. The final model with appropriately constrained and varied paths fit the data well χ^2^(23, 689) = 43.08 *p* = 0.007; SRMR = 0.04; RMSEA = 0.05 (90% CI (0.03, 0.07)); CFI = 0.98, performing slightly worse than the free-varying model, but not significantly with χ^2^_diff_ (9) = 8.05, *p* = 0.529.

## 4. Discussion

Consistent with gateway theory [11], our findings showed there was a significant positive relationship between minor and major substance use for male and female adolescents and also Latino and Black adolescents in a sample from North Carolina. According to gateway theory, when youth use more minor substances (e.g., alcohol, tobacco), they may experiment with and/or progress to harder drugs at a later time. Although our study was cross-sectional, longitudinal studies have found results to support this transition from minor (or gateway) substance use to major substance use [12].

Beyond gateway theory, it is important to note that partial support was found for friends’ delinquency and peer victimization related to minor and major substance use. These findings support social learning and self-derogation theories [17]. Namely, adolescents are more likely to normalize delinquent behaviors (e.g., substance use) if they perceive that their friends engage in substance use and/or these behaviors are positively reinforced by their peers. Furthermore, adolescents are more likely to use substances if they are suffering from low self-esteem brought on by stressful experiences such as victimization by peers. The minor or major substance use could be a coping strategy to deal with the stress or even a way to fit in with other peers. Some ethnic differences are worth highlighting. For example, friends’ delinquency was more strongly related to major substance use by Blacks, while peer victimization was more strongly related to major substance use by Latinos. For Blacks, the social learning theory might be a better explanation, while for Latinos substances might be a way to cope with experiences of victimization.

Next, neighborhood risks were indirectly related to substance use through both peer variables. When adolescents perceive or experience neighborhood risks (e.g., crime and availability of substances), they and their friends have more opportunity to access alcohol, tobacco, cannabis, and major drugs. In addition, when there is more crime and/or substance use in the neighborhood, adolescents are at increased risk of victimization by delinquent peers or neighbors engaging in criminal activities. For girls, exposure to neighborhood risks significantly increased girls’ reports of peer victimization, which in turn, increased girls’ minor substance use. Thus, girls may be especially vulnerable in high risk neighborhoods. 

Lastly, it was found that perceived maternal and paternal monitoring were indirectly and directly related to minor substance use, and indirectly related to major substance use. This finding supports other research showing that parental monitoring can decrease risk of adolescents’ substance use [37,38]. The results also indicated that perceived monitoring by parents can also decrease exposure to neighborhood risks as well as delinquent peers. Thus, when parents are knowledgeable about their children’s activities and whereabouts, then the youth may be less likely to be exposed to risky situations (e.g., crime, delinquency, victimization) and less likely to engage in risky behaviors. In the path models, perceived maternal monitoring was more likely to decrease girls’ minor substance use. However, perceived paternal monitoring was negatively related to friends’ delinquency by boys. Thus, perceived monitoring by mothers and fathers may have differential effects on boys and girls. Ethnic differences were also found. Maternal monitoring of Black youth was more likely to decrease exposure to friends’ delinquency. Black youth are more likely to have a mother at home than father, which may partially explain the ability to monitor associations with delinquent peers. For Latino boys, paternal monitoring was related to exposure to friends’ delinquency. It is possible that in Latino families, fathers are more connected to their sons’ friends.

Some limitations of this study should be noted. The cross-sectional nature of the data does not allow us to establish the directionality of the relationships between the variables. It is possible that some of these relationships are bidirectional. For example, parents may start to monitor their adolescents more if they discover they are engaging in substance use. Future research should use a longitudinal design to better elucidate the temporal priority of these variables. Another limitation is that the current measure for substance use cannot truly capture the wide range of substances available or the degree of use that can occur. Furthermore, data for this study were collected from a single state and were from 9th and 10th grade high school students, thus limiting generalizability. Future studies should replicate this study in different geographic locations and with different age groups. Moreover, the use of self-report surveys for assessing all variables in the study may have inflated relationships due to shared method variance. Future studies might consider using other methods to measure the variables such as actual neighborhood qualities or reports from parents or peers. Since this study only examined parental monitoring, future studies should examine other parenting behaviors or parental styles in relation to neighborhood qualities, peer qualities, and minor and major substance use. Additionally, some participants might have completed the survey in the presence of an adult (e.g., parent); that could have changed responses by the adolescents. Future studies should consider classroom or facility administration, instead of a take-home method. Finally, the sample size impeded examination of differences based on generational statuses or Latino/Black heritages (e.g., Mexican origin versus Salvadoran, African American versus Black). In addition, future studies may also want to consider how personality traits [19] and/or perceptual styles [45] might mediate or moderate the relationships between neighborhood, family, and peer qualities on substance use. 

## Figures and Tables

**Table 1:** Sample characteristics.

	Total	Black Adolescents	Latino Adolescents
N	797	376	421
Age *M*	15.91	16.03	15.81
Grade			
9th grade	30.11%	27.13%	32.78%
10th grade	34.63%	30.59%	38.24%
11th grade	18.82%	16.22%	21.14%
12th grade	16.44%	26.06%	7.84%
Gender			
Boys	45.80%	47.07%	44.66%
Girls	54.20%	52.93%	55.34%
Family structure			
Two-parent intact family	45.17%	23.40%	64.61%
Single-mother family	25.35%	38.83%	13.30%
Stepfather family	18.82%	23.40%	14.73%
Other family forms	10.66%	14.36%	7.36%
Generation status			
First (youth and parents foreign-born)	24.72%	1.33%	45.61%
Second (youth USA-born, at least one parent foreign-born)	24.47%	0.80%	45.61%
Third (youth and parents USA-born)	47.80%	96.01%	4.75%
Birth country of parents			
USA	47.30%	96.01%	3.80%
Mexico	31.37%	0.00%	59.38%
El Salvador	3.76%	0.00%	7.13%
Guatemala	2.13%	0.00%	4.04%
Other country	15.43%	3.99%	25.65%

**Table 2:** Means, standard deviations, and bivariate correlations for boys (above the diagonal) and girls (below the diagonal).

Variables	1	2	3	4	5	6	7
1. Major substance use	---	0.63 **	0.19 **	0.55 **	0.60 **	−0.08	−0.11 *
2. Minor substance use	0.53 **	---	0.30 **	0.35 **	0.70 **	−0.23 **	−0.25 **
3. Neighborhood risks	0.21 **	0.19 **	---	0.19 **	0.34 **	−0.26 **	−0.15 **
4. Peer victimization	0.37 **	0.29 **	0.29 **	---	0.39 **	−0.09	−0.21 **
5. Friends’ delinquency	0.46 **	0.56 **	0.22 **	0.28 **	---	−0.32 **	−0.31 **
6. Mothers’ monitoring	−0.08	−0.30 **	−0.12 *	−0.05	−0.16 **	---	0.50 **
7. Fathers’ monitoring	−0.06	−0.19 **	−0.08	−0.09	−0.12 **	0.31 **	---
Girl youths’ *M*	0.05	0.27	2.01	1.53	0.46	3.40	2.91
Girl youths’ *SD*	0.29	0.56	0.87	0.73	0.66	0.56	0.88
Boy youths’ *M*	0.12	0.33	1.97	1.42	0.54	3.30	2.95
Boy youths’ *SD*	0.43	0.66	0.87	0.70	0.82	0.58	0.83

* *p* < 0.05. ** *p* < 0.01.

**Table 3:** Means, standard deviations, and bivariate correlations for Blacks (above the diagonal) and Latinos (below the diagonal).

Variables	1	2	3	4	5	6	7
1. Major substance use	---	0.60 **	0.19 **	0.46 **	0.60 **	−0.04	−0.00
2. Minor substance use	0.59 **	---	0.23 **	0.36 **	0.62 **	−0.27 **	−0.17 **
3. Neighborhood risks	0.20 **	0.25 **	---	0.20 **	0.27 **	−0.18 **	−0.04
4. Peer victimization	0.44 **	0.25 **	0.30 **	---	0.38 **	−0.08	−0.13 *
5. Friends’ delinquency	0.48 **	0.65 **	0.30 **	0.27 **	---	−0.24 **	−0.11
6. Mothers’ monitoring	−0.14 **	−0.26 **	−0.18 **	−0.05	−0.24 **	---	0.26 **
7. Fathers’ monitoring	−0.19 **	−0.25 **	−0.20 **	−0.13 **	−0.33 **	0.56 **	---
Black youths’ *M*	0.11	0.33	2.00	1.55	0.53	3.37	2.74
Black youths’ *SD*	0.43	0.62	0.94	0.73	0.75	0.58	0.95
Latino youths’ *M*	0.06	0.26	1.96	1.42	0.46	3.34	3.09
Latino youths’ *SD*	0.28	0.59	0.80	0.71	0.72	0.57	0.72

* *p* < 0.05. ** *p* < 0.01.

**Table 4:** Significant path coefficients (unstandardized) for boys/girls.

	Minor Substance Use	Friends’ Delinquency	Peer Victimization	Neighborhood Risks	Maternal Monitoring	Paternal Monitoring
Major substance use	0.20 **	0.09 **	0.10 **			
Minor substance use		0.42 **	0.02/0.10 **		0.01/−0.13 **	−0.05 *
Friends’ delinquency				0.20 **	−0.11 *	−0.24 **/−0.05
Peer victimization		0.20 **		0.04/0.24 **		
Neighborhood risks					−0.29 **	

* *p* < 0.05. ** *p* < 0.01.

**Table 5:** Significant path coefficients (unstandardized) for Blacks/Latinos.

	Minor Substance Use	Friends’ Delinquency	Peer Victimization	Neighborhood Risks	Maternal Monitoring	Paternal Monitoring
Major substance use	0.20 **	0.15 **/0.04 *	0.06 */0.12 **			
Minor substance use		0.43 **	0.07 *		−0.08	−0.04
Friends’ delinquency				0.20 **	−0.18 */−0.002	−0.04/−0.30 **
Peer victimization		0.19 **		0.15 **		
Neighborhood risks					−0.28 **	

* *p* < 0.05. ** *p* < 0.01.

## Data Availability

The data presented in this study, along with SPSS and R syntax, are available on request from the corresponding author.

## Despite Its Limitations

Results of this study suggest several implications for health practitioners and family life educators. For instance, increasing perceptions by adolescents of parental monitoring, especially from mothers, has the possibility of decreasing youths’ involvement in delinquent behaviors. Furthermore, parents can be taught non-intrusive ways to monitor their youth so as to decrease exposure to delinquent peers, high-risk neighborhoods, and illegal substances. Mental health practitioners working with adolescents who are engaging in minor or major substance use should consider interactions with friends, neighborhood environment, and parents in their treatment plan.
